# Effects of Vitamin D Supplementation on IGF-1 and Calcitriol: A Randomized-Controlled Trial

**DOI:** 10.3390/nu9060623

**Published:** 2017-06-17

**Authors:** Christian Trummer, Verena Schwetz, Marlene Pandis, Martin R. Grübler, Nicolas Verheyen, Martin Gaksch, Armin Zittermann, Winfried März, Felix Aberer, Angelika Lang, Claudia Friedl, Andreas Tomaschitz, Barbara Obermayer-Pietsch, Thomas R. Pieber, Stefan Pilz, Gerlies Treiber

**Affiliations:** 1 Department of Internal Medicine, Division of Endocrinology and Diabetology, Medical University of Graz, 8036 Graz, Austria; christian.trummer@medunigraz.at (C.T.); marlene.pandis@medunigraz.at (M.P.); martin.gruebler@gmx.net (M.R.G.); martin.gaksch@gmail.com (M.G.); felix.aberer@medunigraz.at (F.A.); angelika.lang@stud.medunigraz.at (A.L.); barbara.obermayer@medunigraz.at (B.O.-P.); thomas.pieber@medunigraz.at (T.R.P.); stefan.pilz@medunigraz.at (S.P.); gerlies.treiber@medunigraz.at (G.T.); 2 Swiss Cardiovascular Center Bern, Department of Cardiology, Bern University Hospital, 3010 Bern, Switzerland; 3 Department of Internal Medicine, Division of Cardiology, Medical University of Graz, 8036 Graz, Austria; nicolas.verheyen@medunigraz.at (N.V.); andreas.tomaschitz@medunigraz.at (A.T.); 4 Clinic for Thoracic and Cardiovascular Surgery, Heart and Diabetes Center NRW, 32545 Bad Oeynhausen, Germany; azittermann@hdz-nrw.de; 5 Clinical Institute of Medical and Chemical Laboratory Diagnostics, Medical University of Graz, 8036 Graz, Austria; Winfried.Maerz@synlab.com; 6 Department of Internal Medicine, Division of Nephrology, Medical University of Graz, 8036 Graz, Austria; Claudia.Friedl2@klinikum-graz.at; 7 Bad Gleichenberg Clinic, 8344 Bad Gleichenberg, Austria; 8 Medical Clinic V (Nephrology, Hypertensiology, Endocrinology), Medical Faculty Mannheim, Ruperto Carola University Heidelberg, 68167 Mannheim, Germany; 9 CBmed, Center for Biomarker Research in Medicine, 8010 Graz, Austria

**Keywords:** insulin-like growth factor-1, calcitriol, vitamin D supplementation

## Abstract

Increasing evidence suggests a possible interaction between vitamin D and insulin-like growth factor-1 (IGF-1). We aimed to investigate effects of vitamin D supplementation on IGF-1 (primary outcome) and calcitriol (1,25(OH)_2_D) concentrations (secondary outcome). This is a post-hoc analysis of the Styrian Vitamin D Hypertension Trial—a single-center, double-blind, randomized, placebo-controlled trial (RCT) conducted from 2011 to 2014 at the Medical University of Graz, Austria. Two-hundred subjects with arterial hypertension and 25(OH)D concentrations <30 ng/mL were randomized to either receive 2800 IU of vitamin D daily or placebo for eight weeks. A total of 175 participants (mean ± standard deviation age, 60 ± 11 years; 49% women) with available IGF-1 concentrations were included in the present analysis. At baseline, IGF-1 concentrations were significantly correlated with 1,25(OH)_2_D (*r* = 0.21; *p* = 0.005) but not with 25(OH)D (*r* = −0.008; *p* = 0.91). In the RCT, vitamin D had no significant effect on IGF-1 (mean treatment effect 3.1; 95% confidence interval −5.6 to 11.9 ng/mL; *p* = 0.48), but it increased 1,25(OH)_2_D concentrations (mean treatment effect 9.2; 95% confidence interval 4.4 to 13.9 pg/mL; *p* ≤ 0.001). In this RCT, in hypertensive patients with low 25(OH)D concentrations, there was no significant effect of vitamin D supplementation on IGF-1 concentrations. However, we observed a cross-sectional correlation between 1,25(OH)_2_D and IGF-1 and an increase of 1,25(OH)_2_D after vitamin D supplementation.

## 1. Introduction

Vitamin D deficiency is common, and has—apart from its well-known detrimental effects on musculoskeletal health—also been associated with several extraskeletal diseases, such as cardiovascular and immunologic diseases or cancer [1,2,3]. Since many of these outcomes have also been associated with alterations in circulating insulin-like growth factor-1 (IGF-1), several studies have suggested a possible interaction between vitamin D and IGF-1 [4,5,6]. IGF-1 possesses key roles in regulating cellular proliferation and apoptosis as well as energy metabolism, body size, and organ-specific functions [7,8]. It has been observed that IGF-1 concentrations are significantly lower in vitamin D receptor (VDR) knockout mice when compared to wildtype mice [9]. In epidemiological studies, serum concentrations of IGF-1 were positively correlated with 25-hydroxy vitamin D (25(OH)D) [10,11]. Moreover, a small randomized crossover study in healthy men reported that IGF-1 treatment increased free calcitriol—i.e., 1,25-dihydroxyvitamin D (1,25(OH)_2_D)—concentrations, consistent with the hypothesis that IGF-1 may increase the activity of 1-alpha-hydroxylase (the enzyme that converts 25(OH)D to 1,25(OH)_2_D) [12]. On the other hand, vitamin D supplementation may also increase IGF-1 concentrations, but data on this topic are inconsistent. Possible interactions and potential reciprocal effects of vitamin D and IGF-1 are of twofold interest. Firstly, low IGF-1 concentrations are involved in the pathogenesis of detrimental metabolic processes such as disturbed glucose homeostasis, and secondly, vitamin D deficiency is particularly frequent in patients with growth hormone (GH) deficiency, who are usually diagnosed on basis of reduced IGF-1 concentrations [13,14]. In GH deficiency, 25(OH)D is negatively correlated with several cardiometabolic risk factors [15]. 

The present study is a post-hoc investigation of a vitamin D randomized placebo-controlled trial (RCT) in 200 hypertensive patients with low 25(OH)D concentrations and includes cross-sectional and interventional investigations on the relationship between vitamin D and IGF-1. As a primary outcome measure, we evaluated whether vitamin D supplementation as compared to placebo has an effect on IGF-1 serum concentrations. Considering that 1,25(OH)_2_D concentrations may be of particular relevance for the suggested interaction between IGF-1 and 25(OH)D, and in view of a recent meta-analysis showing that vitamin D supplementation increases 1,25(OH)_2_D concentrations [16], we also evaluated the effect of vitamin D on serum concentrations of 1,25(OH)_2_D in our RCT. In addition, we tested whether baseline concentrations of IGF-1 are correlated with 25(OH)D and 1,25(OH)_2_D concentrations.

## 2. Materials and Methods 

### 2.1. Study Design

The current investigation is a post-hoc analysis of the Styrian Vitamin D Hypertension Trial—a single-center, double-blind, placebo-controlled (1:1) parallel-group study performed at the Medical University of Graz, Graz, Austria [17]. The design and methods of this trial have been published [17,18]. The publication of this trial adheres to the Consolidated Standards of Reporting Trials (CONSORT) 2010 statement [19]. The trial was registered at http://www.clinicaltrialsregister.eu (EudraCT number 2009-018125-70) and at http://www.clinicaltrials.gov (ClinicalTrials.gov Identifier NCT02136771). The study protocol was approved by the ethics committee at the Medical University of Graz.

### 2.2. Study Participants 

To be eligible to participate in the study, subjects needed to be 18 years or older with diagnosed arterial hypertension and a serum concentration of 25(OH)D below 30 ng/mL (multiply by 2.496 to convert ng/mL to nmol/L). Arterial hypertension was defined according to published guidelines [20] or by ongoing intake of antihypertensive medication. The exclusion criteria were hypercalcemia (defined as a plasma calcium concentration >2.65 mmol/L), pregnant or lactating women, acute coronary syndrome, cerebrovascular events within the previous two weeks, drug intake as part of another clinical study, an estimated glomerular filtration rate according to the Modification of Diet in Renal Disease formula <15 mL/min per 1.73 m² [21], change of antihypertensive treatment within the previous four weeks or planned changes of antihypertensive treatment, diseases with an estimated life expectancy of less than one year, 24-h systolic blood pressure >160 mmHg or <120 mmHg, 24-h diastolic blood pressure >100 mmHg, any clinically significant acute disease requiring drug treatment, chemotherapy or radiation therapy, or regular intake of >880 IU of vitamin D daily during the last four weeks [17]. The diagnosis of diabetes mellitus was made according to the American Diabetes Association [22], if participants took any antidiabetic medication, or reported to have previously received the diagnosis of diabetes mellitus. Study participants were not instructed to consume a specific diet. According to the Austrian Nutrition Report published in 2012, Austrian women aged 51–64 consume an average of 786 mg of calcium and 108 IU (divide by 40 to convert IU to µg) of vitamin D daily, while men of the same age group consume 802 mg of calcium and 184 IU of vitamin D daily [23].

### 2.3. Intervention

The study medication was filled into numbered bottles according to a computer-generated randomization list. Randomization procedures were conducted using a web-based software (http://www.randomizer.at) with good clinical practice compliance as confirmed by the Austrian Agency for Health and Food Safety. Participants eligible for the study were randomly allocated in a 1:1 ratio to receive either 2800 IU of vitamin D3 as seven oily drops per day (Oleovit D3, Fresenius Kabi Austria, Austria) or a matching placebo as seven oily drops per day for eight weeks. We performed permuted block randomization with a block size of 10 and stratification according to sex. All investigators/authors who enrolled participants, collected data, and assigned intervention were masked to participant allocation.

### 2.4. Outcome Measure

The present study is a post hoc analysis that investigates the between-group differences in IGF-1 (primary outcome) and 1,25(OH)_2_D (secondary outcome) concentrations at study end while adjusting for baseline values [24].

### 2.5. Measurements

Physical examinations, blood sampling, and patient interviews were performed at study visits between 7 and 11 a.m. after an overnight fasting period. The participants then left the hospital for ambulatory blood pressure measurements and 24-h urine collections before they returned to the outpatient department on the following day. On this day, eligible study participants were randomized and started with the intake of the study medication. Serum concentrations of IGF-1, 25(OH)D, and 1,25(OH)_2_D were measured by chemiluminescence immunoassays (Immunodiagnostic Systems Ltd., Boldon, UK) with intra-assay and inter-assay coefficients of variation (CVs) of 2.9–4.2% and 5.4–7.2% (IGF-1); 6.2% and 11.6% (25(OH)D); 6.4–12.1% and 6.6–9.6% (1,25(OH)_2_D), respectively. Details on several other specifics of laboratory measurements have been published [17,25,26,27].

### 2.6. Data Analysis

Information regarding the sample size calculation of the Styrian Vitamin D Hypertension Trial has been published [17]. Continuous data following a normal distribution are shown as means with standard deviations, whereas parameters with a skewed distribution are shown as medians with interquartile ranges. Categorical data are presented as percentages. Where appropriate, skewed variables were log(e) transformed before they were used in parametric analyses. Comparisons between the vitamin D and placebo group at baseline were calculated by the unpaired Student *t*-test, the Mann–Whitney-U-test, or chi-square test. Differences across quartiles of IGF-1 concentrations at baseline were calculated by analysis of variance (ANOVA) with *p* for trend or by chi-square test. Pearson correlation analysis was performed to evaluate the correlation of baseline concentrations of IGF-1 with 25(OH)D and 1,25(OH)_2_D concentrations. Comparisons between baseline and follow-up concentrations of 25(OH)D were calculated by the paired Student *t*-test. Analysis of covariance with adjustments for baseline values was used to test for differences in the outcome variables (i.e., IGF-1 and 1,25(OH)_2_D) between the treatment and the placebo group at follow-up visit [24]. Analyses were performed according to the intention-to-treat principle with no data imputation and inclusion of all participants with baseline and follow-up values of the respective outcome variable. A *p*-value < 0.05 was considered statistically significant. All statistical operations were performed with SPSS version 23 (SPSS, Chicago, IL, USA).

## 3. Results

Of approximately 1700 persons who were invited to participate in the study, 518 gave written informed consent and were assessed for eligibility, of whom 200 were randomized and 188 completed the trial. Randomization and follow-up visits were performed between June 2011 and August 2014. The current study is restricted to randomized study participants with available IGF-1 concentrations at baseline and study end (*n* = 175).

Baseline characteristics of the randomized participants are shown in Table 1. At baseline, concentrations of 25(OH)D were significantly lower in the placebo group when compared to the vitamin D group. The remaining baseline characteristics did not show any significant differences between the study groups (Table 1). At baseline, 37 (21.1%) participants showed parathyroid hormone (PTH) concentrations above the upper limit of the reference range (i.e., 65.0 pg/mL), suggesting secondary hyperparathyroidism. Despite rigorous monitoring, one participant was randomized with an elevated serum calcium concentration of 2.69 mmol/L, and therefore violated the exclusion criteria of hypercalcemia. Nevertheless, by adhering to the intention-to-treat principle, we did not exclude this participant from our final analyses.

Baseline characteristics according to quartiles of IGF-1 concentrations are shown in Table 2. We observed a significant trend across the quartiles for age, body-mass-index (BMI), prevalence of diabetes mellitus, fasting glucose, hemoglobin A1c (HbA1c), and C-reactive protein concentrations (Table 2). When participants were stratified into quartiles by their baseline 25(OH)D concentrations, we observed a significant trend for diabetes mellitus diagnoses toward lower 25(OH)D concentrations, as well as a concomitant trend in HbA1c (Table S1). In Pearson correlation analysis, IGF-1 was significantly correlated with serum concentrations of 1,25(OH)_2_D (*r* = 0.21, *p* = 0.005), but not with serum concentrations of 25(OH)D (*r* = −0.008, *p* = 0.91).

After intervention, 25(OH)D concentrations increased from 22.1 ± 5.4 ng/mL to 36.3 ± 7.4 ng/mL in the vitamin D group (*p* ≤ 0.001). In the placebo group, 25(OH)D also slightly increased from 20.3 ± 5.7 ng/mL to 23.7 ± 9.1 ng/mL (*p* ≤ 0.001). Vitamin D supplementation did not have a significant effect on concentrations of IGF-1, but there was a significant increase in 1,25(OH)_2_D in the vitamin D group (Table 3). Our results did not materially change in subgroups with baseline 25(OH)D concentrations below 16 and 20 ng/mL, respectively (Table S2).

## 4. Discussion and Conclusions

In this RCT, in hypertensive patients with low 25(OH)D concentrations, there was no significant effect of vitamin D supplementation on IGF-1 concentrations. While there was no significant correlation between IGF-1 and 25(OH)D, we observed a significant correlation between IGF-1 and 1,25(OH)_2_D and a significant increase of 1,25(OH)_2_D in the vitamin D group when compared to the placebo group.

The main finding of our study (i.e., that we did not observe an effect of vitamin D supplementation on IGF-1 concentrations) is in line with a previous RCT in 318 overweight and obese subjects, who received 40,000 or 20,000 IU of cholecalciferol per week for one year [28]. In this RCT by Kamycheva et al. [28], there was no effect of vitamin D on IGF-1 concentrations, but in a subgroup of non-severely obese participants (BMI < 35 kg/m²), vitamin D treatment lowered the IGF-1/IGF binding protein-3 (IGFBP-3) ratio—a measure of free IGF-1 concentrations. Another study in Latino and African-American subjects with pre-diabetes and hypovitaminosis D also did not show any effect of vitamin D supplementation on IGF-1 [29]. By contrast, a few previous uncontrolled or unblinded studies reported an increase in IGF-1 concentrations after vitamin D supplementation [30,31,32]. Differences in study populations and study design may have accounted for these inconsistencies, but the findings of our study along with previous ones [28,29] strongly argue against a clinically relevant effect of vitamin D supplementation on IGF-1 concentrations. This is further supported by the fact that we found no correlation between IGF-1 concentrations and 25(OH)D at baseline. Previous clinical studies on the association between 25(OH)D and IGF-1 showed mixed results with positive, negative, or no significant correlation [10,28,33]. Therefore, the relationship between vitamin D and IGF-1 is still puzzling.

Possible pathophysiological relationships between vitamin D and IGF-1 are still not clear. The major source of circulating IGF-1 is the liver, which is considered a target tissue for vitamin D [34,35,36]. Although VDR knockout mice produce higher IGF-1 concentrations compared to controls and some experimental data suggest increased IGF-1 expression induced by VDR activation [9,37], there seems to be no clinically relevant effect of vitamin D supplementation on IGF-1 concentrations, as evidenced by our trial. On the other hand, there may be an effect in the opposite direction (i.e., that IGF-1 modulates vitamin D concentrations and their metabolism). Data from several studies indicate a possible induction of renal 1α-hydroxylase by IGF-1, whereas there seems to be no effect of IGF-1 on 25-hydroxylation [34,38,39,40,41]. This fits well to our results of a positive correlation between IGF-1 and 1,25(OH)_2_D, the product of 1-alpha-hydroxylase, but of a lack of a significant correlation between IGF-1 and 25(OH)D. In line with this, Kamenický et al. [41] found signficantly higher concentrations of 1,25(OH)_2_D in patients with active acromegaly when compared to subjects with controlled disease. Therefore, and in view of a small study in healthy men suggesting that IGF-1 increases free 1,25(OH)_2_D concentrations [12], there is still a need to further evaluate the impact of GH/IGF-1 treatment on the vitamin D system.

Another major finding of our trial is the significant increase of 1,25(OH)_2_D after vitamin D supplementation. These results are in line with a recent meta-analysis by Zittermann et al. [16], who reported an increase in concentrations of 1,25(OH)_2_D after vitamin D supplementation by 12.2 pmol/L (5.1 pg/mL) or 18.8 pmol/L (7.8 pg/mL), if only studies with a low risk of bias were considered. The increase of 1,25(OH)_2_D after vitamin D supplementation may be of clinical significance because 1,25(OH)_2_D is the so-called active vitamin D hormone with the highest affinity for the VDR, and is inversely associated with mortality [42,43].

A limitation of our study is that our results are derived from patients with hypertension and low 25(OH)D concentrations, and our findings are therefore not readily generalizable to other study populations that are of potential interest for the relationship between IGF-1 and vitamin D (e.g., patients with GH deficiency or acromegaly). Additionally, the nature of our study as a post hoc analysis warrants some caution regarding data interpretation. Furthermore, our study lacks a more detailed assessment of the GH/IGF-1 axis, such as measurements of IGF binding proteins or GH itself, but IGF-1 is a well-established parameter for the assessment of the GH/IGF-1 system. A clear strength of our study is its design as a RCT and the confirmation of an effective vitamin D treatment by a significant increase in 1,25(OH)_2_D concentrations, and as already shown in previous publications of this RCT [17], by a significant treatment effect on 25(OH)D and PTH concentrations. The validity of our data is further underscored by the confirmation of well-known associations of IGF-1 concentrations with, for example, BMI, parameters of glucose homeostasis, or age [14,44,45]. Finally, our findings significantly add to the existing data on the relationship between IGF-1 and vitamin D, as our study is—apart from two previous RCTs—the only RCT on this topic in a well-characterized population including measurements of 1,25(OH)_2_D.

In conclusion, we did not observe an effect of vitamin D supplementation on IGF-1 concentrations in patients with vitamin D insufficiency and arterial hypertension. However, as a secondary outcome parameter, we found a significant increase in concentrations of 1,25(OH)_2_D following vitamin D supplementation and a significant cross-sectional correlation between 1,25(OH)_2_D and IGF-1. Further studies are still warranted to evaluate the relationship between vitamin D and IGF-1/GH in patients with GH deficiency and acromegaly and to clarify whether treatment with IGF-1/GH has a relevant effect on vitamin D metabolites.

## Figures and Tables

**Table 1:** Baseline characteristics of all randomized participants with available IGF-1 values at baseline and follow-up.

	All (*n* = 175)	Vitamin D (*n* = 86)	Placebo (*n* = 89)	*p*-Value
Age (years)	60 ± 11	61 ± 11	60 ± 12	0.68
Females (%)	49	47	51	0.59
BMI (kg/m²)	30.2 ± 5.1	30.6 ± 4.5	29.8 ± 5.7	0.30
Office systolic BP (mmHg)	143 ± 15	143 ± 15	143 ± 15	0.72
Office diastolic BP (mmHg)	87 ± 10	87 ± 10	88 ± 10	0.52
IGF-1 (ng/mL)	118.0 ± 42.1	113.4 ± 38.2	122.5 ± 45.2	0.16
25(OH)D (ng/mL)	21.2 ± 5.6	22.1 ± 5.4	20.3 ± 5.7	0.04
1,25(OH)_2_D (pg/mL)	50.5 ± 19.0	52.1 ± 18.6	49.0 ± 19.3	0.29
PTH (pg/mL)	49.1 (39.3–63.6)	49.0 (39.3–61.9)	51.3 (39.1–65.6)	0.83
Serum calcium (mmol/L)	2.36 ± 0.10	2.37 ± 0.10	2.36 ± 0.11	0.92
Serum phosphate (mmol/L)	0.95 ± 0.16	0.93 ± 0.17	0.98 ± 0.15	0.30
Diabetes mellitus (%)	35	30	40	0.16
Fasting glucose (mg/dL)	100 (91–130)	101 (94–124)	98 (89–140)	0.66
HbA1c (mmol/mol)	40 (37–50)	40 (36–47)	40 (37–51)	0.41
HOMA-IR	2.01 (1.18–3.79)	2.07 (1.24–3.93)	1.79 (1.12–3.75)	0.997
eGFR (mL/min/1.73m²)	78.9 ± 18.3	80.4 ± 18.6	77.4 ± 17.9	0.28
Triglycerides (mg/dL)	120 (78–163)	123 (80–168)	118 (73–161)	0.82
HDL-cholesterol (mg/dL)	57 ± 16	56 ± 16	58 ± 17	0.32
LDL-cholesterol (mg/dL)	113 ± 40	115 ± 42	112 ± 40	0.63
CRP (mg/L)	1.8 (0.9–3.6)	2.2 (0.9–3.9)	1.4 (0.8–3.0)	0.06
Use of calcium supplements (%)	9	11	8	0.61
Use of additional vitamin D supplements (%)	8	6	10	0.41

Data are shown as means with standard deviation, median, and interquartile range, or as percentages, whichever was appropriate. Comparisons between the groups (vitamin D vs. placebo) were calculated by Student *t*-test, Mann–Whitney-U-test, or chi-square test. BMI = body-mass index; BP = blood pressure; IGF-1 = insulin-like growth factor-1; 25(OH)D = 25-hydroxyvitamin D; 1,25(OH)_2_D = 1,25-dihydroxyvitamin D; PTH = parathyroid hormone; HbA1c = hemoglobin A1c; HOMA-IR = homeostasis model assessment-insulin resistance; eGFR = estimated glomerular filtration rate; HDL-cholesterol = high-density lipoprotein-cholesterol; LDL-cholesterol = low-density lipoprotein-cholesterol; CRP = C-reactive protein.

**Table 2:** Baseline characteristics of participants after partition in quartiles according to IGF-1 concentrations.

	IGF-1 Quartiles				
	Quartile 1 ≤89.5 ng/mL (*n* = 44)	Quartile 2 89.6–111.0 ng/mL (*n* = 44)	Quartile 3 111.1–143.0 ng/mL (*n* = 44)	Quartile 4 ≥143.1 ng/mL (*n* = 43)	*p*-Value for Trend
Age (years)	62 ± 8	62 ± 8	60 ± 11	57 ± 16	0.02
Females (%)	57	48	48	42	0.19
BMI (kg/m²)	32.0 ± 6.2	30.2 ± 4.0	30.3 ± 5.0	28.2 ± 4.4	0.001
Office Systolic BP (mmHg)	146 ± 15	142 ± 15	145 ± 15	139 ± 15	0.08
Office Diastolic BP (mmHg)	87 ± 10	87 ± 10	87 ± 11	88 ± 10	0.86
25(OH)D (ng/mL)	19.7 ± 6.0	22.1 ± 5.7	21.9 ± 5.1	20.9 ± 5.4	0.35
1,25(OH)_2_D (pg/mL)	47.9 ± 20.1	48.3 ± 17.3	52.5 ± 19.5	53.6 ± 18.9	0.10
PTH (pg/mL) *	55.2 (39.7–70.2)	44.0 (35.6–55.5)	53.1 (42.3–63.4)	48.1 (38.5–63.4)	0.94
Serum calcium (mmol/L)	2.36 ± 0.10	2.37 ± 0.11	2.36 ± 0.11	2.37 ± 0.10	0.87
Serum phosphate (mmol/L)	0.96 ± 0.14	0.96 ± 0.17	0.94 ± 0.18	0.96 ± 0.15	0.91
Diabetes mellitus (%)	52	34	25	30	0.02
Fasting glucose (mg/dL) *	121 (98–169)	100 (92–134)	96 (92–106)	94 (88–122)	<0.001
HbA1c (mmol/mol) *	44 (38–57)	41 (38–56)	39 (36–45)	40 (36–47)	0.006
HOMA-IR *	2.49 (1.43–6.22)	2.11 (1.42–3.44)	1.72 (1.14–4.12)	1.53 (1.03–3.03)	0.23
eGFR (mL/min/1.73m²)	77.0 ± 20.1	80.0 ± 15.1	78.4 ± 18.7	80.1 ± 19.2	0.54
Triglycerides (mg/dL) *	116 (81–153)	128 (83–185)	126 (71–180)	116 (68–135)	0.46
HDL-cholesterol (mg/dL)	56 ± 13	58 ± 20	57 ± 19	57 ± 13	0.94
LDL-cholesterol (mg/dL)	104 ± 45	112 ± 42	120 ± 44	118 ± 30	0.08
CRP (mg/L) *	2.2 (1.0–4.9)	2.0 (1.2–3.8)	1.3 (0.9–2.6)	1.5 (0.6–3.8)	0.009

Data are shown as means with standard deviation, median, and interquartile range, or as percentages, whichever was appropriate. *p*-values for trends were calculated by ANOVA or chi-square test, whichever was appropriate. * Skewed variables for which logarithmic transformed values were used in ANOVA but untransformed values are shown in the table.

**Table 3:** Selected parameters at baseline and follow-up in study participants with available values at both study visits.

	Baseline	Follow-Up	Treatment Effect	*p*-Value
IGF-1 (ng/mL)				
Vitamin D (*n* = 86)	113.4 ± 38.2	116.9 ± 33.3	3.1 (−5.6 to 11.9)	0.48
Placebo (*n* = 89)	122.5 ± 45.2	120.0 ± 47.6		
1,25(OH)_2_D (pg/mL)				
Vitamin D (*n* = 85)	52.1 ± 18.6	60.0 ± 22.2	9.2 (4.4 to 13.9)	<0.001
Placebo (*n* = 86)	49.0 ± 19.3	48.7 ± 16.6		

Data are shown as means with standard deviation. Treatment effects with 95% confidence interval and *p*-values were calculated by ANCOVA for group differences at follow-up with adjustment for baseline values.

## Supplementary Materials

The following are available online at http://www.mdpi.com/2072-6643/9/6/623/s1. Table S1: Baseline characteristics of participants after partition in quartiles according to 25-hydroxyvitamin D concentrations; Table S2: IGF-1 and 1,25(OH)_2_D at baseline and follow-up in subgroups with 25(OH)D concentrations below 20 and 16 ng/mL at baseline.
