# Supplementary material for: Effects of Vitamin D Supplementation on IGF-1 and Calcitriol: A Randomized-Controlled Trial
Source: Nutrients. 2017 Jun 17;9(6):623. doi: 10.3390/nu9060623 (PMC5490602; doi:10.3390/nu9060623)
Supplement: Supplementary file 1 [file nutrients-09-00623-s001.pdf]

Table S1. Baseline characteristics of participants after partition in quartiles according to 25-hydroxyvitamin D concentrations.

	25-hydroxyvitamin D quartiles				
	**Quartile 1 ≤17.2 ng/mL (*n* = 44)**	**Quartile 2 17.3-21.9 ng/mL (*n* = 44)**	**Quartile 3 22.0-25.7 ng/mL (*n* = 46)**	**Quartile 4 ≥25.8 ng/mL (*n* = 41)**	***p*-value for trend**
Age [years]	61 ± 11	59 ± 10	60 ± 12	61 ± 12	0.85
Females [%]	43	50	57	44	0.76
BMI [kg/m²]	30.2 ± 5.3	30.8 ± 6.0	30.1 ± 4.9	29.7 ± 4.2	0.51
Office systolic BP (mmHg)	145 ± 16	142 ± 13	142 ± 16	143 ± 16	0.56
Office diastolic BP (mmHg)	86 ± 10	89 ± 11	88 ± 10	85 ± 10	0.73
IGF-1 [ng/mL]	120.7 ± 52.4	120.2 ± 41.2	115.7 ± 38.9	115.4 ± 34.5	0.48
1,25(OH)$_2$D [pg/mL]	45.3 ± 18.4	49.9 ± 19.2	56.1 ± 20.8	50.7 ± 16.0	0.09
PTH [pg/mL]*	54.3 (40.3-65.1)	52.8 (36.3-64.1)	47.8 (39.8-66.6)	45.9 (39.2-55.0)	0.19
Serum calcium [mmol/L]	2.26 ± 0.08	2.26 ± 0.10	2.26 ± 0.11	2.29 ± 0.11	0.19
Serum phosphate [mmol/L]	0.94 ± 0.16	0.96 ± 0.17	0.94 ± 0.16	0.96 ± 0.16	0.77
Diabetes mellitus [%]	57	25	30	29	0.02
Fasting glucose [mg/dL]*	116 (91-158)	96 (93-174)	99 (90-117)	100 (93-127)	0.10
HbA1c [mmol/mol]*	47 (38-59)	39 (37-42)	40 (36-45)	40 (36-47)	0.01
HOMA-IR*	2.54 (1.29-5.68)	1.78 (1.15-3.89)	1.56 (1.08-2.57)	2.27 (1.18-4.50)	0.24
eGFR [mL/min/1.73m²]	80.2 ± 19.8	79.1 ± 16.7	76.8 ± 16.2	79.6 ± 20.7	0.74
Triglycerides [mg/dL]*	117 (75-185)	120 (86-163)	117 (69-153)	124 (75-163)	0.85
HDL-cholesterol [mg/dL]	56 ± 14	56 ± 15	58 ± 16	56 ± 21	0.76
LDL-cholesterol [mg/dL]	109 ± 45	121 ± 41	116 ± 36	107 ± 42	0.71
CRP [mg/L] *	1.6 (0.9-3.0)	1.5 (0.8-3.3)	1.7 (0.9-4.1)	2.0 (1.2-3.6)	0.31

Data are shown as means with standard deviation, median and interquartile range, or as percentages, whichever was appropriate. P-values for trends were calculated by ANOVA or chi-square test, whichever was appropriate. *Skewed variables for which logarithmic transformed values were used in ANOVA but untransformed values are shown in the Table. BMI = body-mass index, BP = blood pressure, IGF-1 = insulin-like growth factor-1, 1,25(OH)$_2$D = 1,25-dihydroxyvitamin D, PTH = parathyroid hormone, HbA1c = hemoglobin A1c; HOMA-IR = homeostasis model assessment-insulin resistance, eGFR = estimated glomerular filtration rate, HDL-cholesterol = high-density lipoprotein-cholesterol, LDL-cholesterol = low-density lipoprotein-cholesterol, CRP = C-reactive protein.

Table S2. IGF-1 and 1,25(OH)$_2$D at baseline and follow-up in subgroups with 25(OH)D concentrations below 20 and 16 ng/mL at baseline.

		**Baseline**	**Follow-Up**	**Treatment Effect**	***p*-value**
**IGF-1**	25(OH)D <20 ng/mL (*n* = 70)				
	Vitamin D (*n* = 29)	114.8 ± 49.2	117.4 ± 37.4	3.6 (−13.2 to 20.5)	0.67
	Placebo (*n* = 41)	124.9 ± 52.8	120.9 ± 57.4		
	25(OH)D <16 ng/mL (*n* = 39)				
	Vitamin D (*n* = 14)	109.3 ± 56.7	112.5 ± 32.2	9.1 (−16.6 to 34.7)	0.48
	Placebo (*n* = 25)	123.3 ± 46.7	112.1 ± 55.0		
**1,25(OH)$_2$D**	25(OH)D <20 ng/mL (*n* = 67)				
	Vitamin D (*n* = 29)	49.2 ± 16.1	59.3 ± 23.2	10.0 (1.8 to 18.2)	0.02
	Placebo (*n* = 38)	44.1 ± 19.7	45.5 ± 16.6		
	25(OH)D <16 ng/mL (*n* = 37)				
	Vitamin D (*n* = 14)	50.9 ± 15.9	68.1 ± 27.0	14.8 (2.0 to 27.7)	0.03
	Placebo (*n* = 23)	41.0 ± 17.8	44.3 ± 17.2		

Data are shown as means with standard deviation. Treatment effects with 95% confidence interval and p-values were calculated by ANCOVA for group differences at follow-up with adjustment for baseline values. IGF-1 = insulin-like growth factor-1, 1,25(OH)$_2$D = 1,25-dihydroxyvitamin D, 25(OH)D = 25-hydroxyvitamin D.
